# Supplementary material for: Physiologic and molecular response of Fusarium oxysporum f. sp. zingiberi to ginger autotoxins
Source: PLoS One. 2026 May 28;21(5):e0339014. doi: 10.1371/journal.pone.0339014 (PMC13218467; doi:10.1371/journal.pone.0339014)
Supplement: Attachment — Submitted filename: PONE-D-25-63719_Review report.docx [file pone.0339014.s007.docx]

I have carefully reviewed the manuscript entitled “*In vitro* Study of the Growth, Reproduction, and Pathogenicity Responses of *Fusarium oxysporum* f. sp. *zingiberi* to Autotoxins from Ginger.” This study is relevant and of potential interest, as it demonstrates that four ginger-related autotoxins differentially affect the growth, reproduction, and virulence-associated traits of *Fusarium oxysporum*, thereby contributing to *Fusarium* wilt under long-term ginger monoculture. The work further provides mechanistic insight into how these autotoxins may influence disease development. Overall, the manuscript is within the scope of the journal. However, the presentation and writing of the manuscript require substantial improvement, and several important concerns should be addressed before the manuscript can be considered for publication.

Line 1-2 It is recommended that the title be revised to more clearly convey the key message and core theme of the study. The title should present the main focus of the work at a single glance, without requiring the reader to refer to the main text for clarification. In addition, *in vitro* should be italicized in the title. At present, the word “study” is too general and does not clearly reflect the specific message or contribution of the work.

Line 10 The email address of the corresponding author appears to be incorrect. Please provide the correct email address, including the proper “@” symbol and domain name, to ensure accuracy.

Line 23-39 The abstract should be revised to include a clearer problem statement, the existing research gap, and the need or importance of this study in addressing that gap, while more explicitly highlighting the novelty and innovation of the research. In addition, there appears to be an inconsistency in the pathogen name: the title refers to *Fusarium oxysporum* f. sp. *zingiberi*, whereas line 24 mentions *Fusarium oxysporum* f. sp. *niveum* and abbreviates it as Foz. Please verify and correct this for accuracy and consistency throughout the manuscript. The treatment information is also unclear. The term “representative autotoxins” should be defined more precisely, as its meaning is not evident in the current context. Similarly, since the title refers to autotoxins from ginger, it should be clarified whether these compounds were directly extracted from ginger, naturally present in ginger and then applied experimentally, or artificially prepared. The concentrations used in each treatment should also be clearly reported. Furthermore, the result section would benefit from reorganization in a more systematic and mechanistic manner, so that the findings present a clear progression rather than appearing randomly arranged. The results should lead to clear concluding statements that are well aligned with the study objectives, support the title, and emphasize the novelty of the work. Finally, it would strengthen the manuscript to include future perspectives, particularly regarding the scientific significance of the findings and their potential practical benefits for farmers.

Line 99-231 It is suggested that the contents be arranged in a more systematic, stepwise manner so that the methodological details are presented clearly from beginning to end, ensuring reproducibility. You mention that the autotoxins used in this study have been documented previously; if so, the novelty of the present work should be clarified. Please indicate whether these compounds were selected based on prior reports and newly evaluated in a different context, or whether they were simply designated as autotoxins in this study. This point needs to be explained more clearly. In addition, the Table 1 should focus only on the details directly relevant to the present experiment, rather than including doses from other experiments, as this may create confusion. If those doses were optimized in a previous study, there is no need to report the additional concentrations here unless they are directly relevant to the current work. Furthermore, in the methods section, the subsection headings should be written according to methodological procedures rather than beginning with phrases such as “effects of autotoxins on different parameters,” which are more appropriate for the results section. Revising the subsection headings to reflect the actual experimental methods would improve the clarity and organization of the manuscript.

Line 232-367 In the results section, the subheadings should be revised to be concise, informative, and sufficiently descriptive of the main content presented under each subsection. Clear and well-structured subheadings would improve the organization of the section and help guide the reader through the key findings more effectively.

Line 368 503 The discussion section would benefit from being divided into clearly defined subsections, with the content organized under appropriate headings for improved clarity and structure. This section should emphasize the major findings of the study and provide a deeper interpretation of the morphological, biochemical, physiological, and molecular mechanisms underlying the observed responses. Moreover, the conclusion should be presented as a separate and independent section, as commonly required by many journals. In the present manuscript, the conclusion appears to be incorporated within the discussion section (lines 491–503), and this should be revised in accordance with the journal’s formatting guidelines. The conclusion section should summarize the main findings in a systematic and mechanistic manner and lead to a clear, strong concluding statement that directly supports the study objectives and research hypothesis, while remaining well aligned with the title of the manuscript. In addition, the authors are encouraged to acknowledge the limitations of the study, outline future research perspectives, and highlight the practical significance of the work for both the scientific community and farmers.

Line 505-693 The references should be thoroughly checked and revised to ensure full compliance with the journal’s formatting guidelines. At present, there are several inconsistencies in reference style. For instance, the journal name appears in uppercase letters in lines 517–518, while journal names are abbreviated in most other entries; however, in line 564, the journal name is provided in full. These inconsistencies should be corrected to maintain accuracy, uniformity, and adherence to the journal’s required format.
