# Supplementary material for: Physiologic and molecular response of Fusarium oxysporum f. sp. zingiberi to ginger autotoxins
Source: PLoS One. 2026 May 28;21(5):e0339014. doi: 10.1371/journal.pone.0339014 (PMC13218467; doi:10.1371/journal.pone.0339014)
Supplement: Attachment — Submitted filename: Response to Reviewers.docx [file pone.0339014.s009.docx]

**Dear editor and reviewers,**

Please find attached our revised manuscript (PONE-D-25-63719) along with detailed responses to the reviewers’ comments. We sincerely thank the reviewers for their valuable and constructive feedback, which was instrumental in improving the manuscript. Our point-by-point responses to each comment are provided below. We hope that the revised manuscript meets the journal’s standards and is considered suitable for publication.

**Journal Requirements:**

Summary

**1.Please ensure that your manuscript meets PLOS ONE's style requirements, including those for file naming.**

Thank you. We have revised the manuscript to align with the journal's style guidelines. We believe that the present manuscript now adheres to the format requirements.

**2.** **We note that the grant information you provided in the ‘Funding Information’ and ‘Financial Disclosure’ sections do not match.** **When you resubmit, please ensure that you provide the correct grant numbers for the awards you received for your study in the ‘Funding Information’ section.**

We apologize for any confusion caused. We have rectified the grant information in the relevant sections and verified the grant numbers. This information is now included in the financial disclosure section.

**3. Thank you for stating the following financial disclosure: “This work was supported by grants from the Shandong Provincial Natural Science Foundation (grant number: ZR2023QC326) and Key R&D Program of Shandong Province，China (grant number: 2023TZXD028). Li received the first project and Xu received the second project.” Please state what role the funders took in the study. If the funders had no role, please state: "****The funders had no role in study design, data collection and analysis, decision to publish, or preparation of the manuscript." If this statement is not correct you must amend it as needed. Please include this amended Role of Funder statement in your cover letter; we will change the online submission form on your behalf.**

Thank you for your suggestion. We have corrected the information in the Financial Disclosure section. Now this description is “*This study was funded by the Shandong Provincial Natural Science Foundation (ZR2023QC326), China, the Key R&D Program of Shandong Province, China (2023TZXD028). The funders had no role in study design, data collection and analysis, decision to publish, or preparation of the manuscript.*”

**4. We notice that your supplementary figures are uploaded with the file type 'Figure'. Please amend the file type to 'Supporting Information'. Please ensure that each Supporting Information file has a legend listed in the manuscript after the references list.**

We apologize for any confusion caused. We have revised the file type to "Supporting Information" and confirm that each file includes its own legend, which is listed in the manuscript following the references section.

**5. If the reviewer comments include a recommendation to cite specific previously published works, please review and evaluate these publications to determine whether they are relevant and should be cited. There is no requirement to cite these works unless the editor has indicated otherwise.**

Thank you. We will review and evaluate these suggestions whether the previous works should be cited. If there was no necessity to cite, we will not do this work.

**Reviewers’ comment**

**Reviewer #1**

Summary

The article entitled “In vitro study of the growth, reproduction and pathogenicity responses of *Fusarium oxysporum* f. sp. *zingiberi* to autotoxins from ginger” resulted to me quite interesting well written but, the conclusions does not fit with the results presented and I am not according to them, this makes me to suggesting several points that might be improved after an extensive review of content, conclusions, discussion and some changes that might be useful to improve the scientific impact and general quality of the article and conclusions obtained.

**Title:**

**1. I would focus the title adjusting it to the main conclusions of the article, from my point of view I see much more relevant the founding about the effects of the different metabolites into the fungal metabolism so I would suggest this kind of title or similar: Physiologic and molecular response of *Fusarium oxysporum* f. sp. *zingiberi* to ginger specific metabolites.” I would suggest to author to review first my suggestion to understand this proposed title, based principally into the quite differences found between the effects produced by the different plant metabolites, more than the term autotoxins, considering that the specific effects of plant metabolites against high impact phytopathogens is poor and scarce known compared to other areas of knowledge.**

We appreciate your insightful suggestions. The primary objective of this study was to elucidate a common natural phenomenon: the reason why extending the cropping period leads to more severe outbreaks of plant diseases. Building on previous research, it has been suggested that certain autotoxic chemicals can affect the physiological activities of pathogens in various crops. Consequently, this study was conducted based on this hypothesis, aiming to interpret and comprehend the responses of *Fusarium oxysporum* f. sp. *zingiberi* to ginger metabolites. Therefore, the phrase "the physiological and molecular response of *Foz*" more accurately encapsulates the focus of this study.

We also considered that plant metabolites serve a protective function against pathogen infection in plants, and the specific effects of these metabolites on high-impact phytopathogens remain inadequately understood. Notably, the compounds utilized in the present study not only affected pathogen activities but also inhibited the growth of ginger, as extensively documented in a previous study (Han et al., 2012a). Consequently, we propose that the term "autotoxins" is more appropriate than "specific metabolites." In conclusion, we have revised the title of the present study to "**Physiologic and Molecular Response of *Fusarium oxysporum* f. sp. *zingiberi* to Ginger Autotoxins.**" We believe that this title accurately reflects the scope and findings of the study.

**Abstract:**

**2. Considering making a big change into the structure and content of the abstract, check line 68 to 75 to present the results under this point of view, add all detailed results including FUB3 and FUB9 not included, highlight the role of coumarin but consider that it might be possible that the fungus is defending from coumarin effect, trying to not death and this might be the reason because you see the inhibition of mycelial growth but strong mycotoxin synthesis, because in plant the fungus try to eat death tissues and it likes a defense response of the fungus against coumarin more than that this metabolite acts as an autotoxin to promote Foz. Explain also at the abstract in which the autotoxins are representative, but I would make a great effort for differentiate what is a fungal defense response against toxic and plant-defense-metabolites such as coumarin and derivates and which produce an effect of real autotoxin, which does not fit with current plant defense responses.**

We appreciate your valuable suggestions. We have substantially revised the content of the Abstract to emphasize the defensive role of autotoxins. Our findings suggest that plants modulate the growth, reproduction, and pathogenicity of pathogens by secreting extracellular products, whereas pathogens counteract this regulation by upregulating pathogenicity factors. Consequently, we have drawn new conclusions for the present study and introduced novel perspectives in the Abstract section. Additionally, we have addressed the representativeness of the autotoxins utilized in the current study, as indicated in **line 29, page 2**. We believe that this revised version communicates our findings more accurately and effectively.

**3. Lines 28-31.- make a fussion and shorter sentence which is repetitive.**

We have integrated these sentences and employed shorter, more concise sentences to articulate this conclusion. We believe that our current description fulfills this requirement.

**4. Line 34-39.- consider change the conclusions accordingly to results.**

We appreciate your suggestions. Based on these suggestions, we derived new conclusions and redefined the primary perspectives as follows: plant metabolites inhibit the growth, reproduction, and virulence of phytopathogens. However, pathogens counteract plant responses by upregulating pathogenicity factors. In this study, we emphasized the role of plant metabolites in defense, and we believe that the current conclusions effectively elucidate our findings.

**Introduction**
**5. Lines 53-54.- detail, is there varieties,** **ecotypes with different resistances? Explain here.**

Thank you. We have added some sentences to introduce this information in **line 55, page 3**. “*Owing to the scarcity of viable seeds and limited genetic diversity, breeding for disease resistance presents significant challenges. Although certain resistant cultivars have been developed and implemented, Fusarium yellow continues to be prevalent across Asia, North America, and Oceania.”*

**6. Line 62.-** **cite roots here.**

Thank you. We have incorporated sentences to introduce the potential sources of pathogens and the symptoms exhibited by infected plants to ensure coherence throughout the manuscript (**line 68, page 4**). "*Infected plants will exhibit chlorosis of their leaves, ultimately leading to the wilting and death of the entire above-ground portion of the plant. The wilted tissues will fall to the soil, where they will be decomposed by microorganisms, subsequently serving as potential sources for the accumulation of pathogens.*"
**7. Lines 68-75.- consider add this part to abstract.**

Thank you for your suggestions. We have revised this section for conciseness and incorporated it into the abstract on **line 24, page 2**.
**8. Line 82.- explain why coumarin inhibit plant growth? Or is just that coumarin is produced in response to the pathogen and plant stop the growth until defense mechanism are activated? Justify. Currently coumarin is produced as plant-defense metabolite.**

We apologize for any confusion. While both cinnamic acids and coumarins can inhibit plant growth, their underlying mechanisms may differ. Cinnamic acid, a specific phenolic acid, not only directly inhibits seedling growth but also induces the physiological activities of phytopathogens. Conversely, coumarins may stimulate conidial germination and mycotoxin production by phytopathogens, thereby inhibiting plant growth. Consequently, describing these compounds together may be inappropriate. We have therefore revised certain sentences for greater clarity in **line 95, page 5**. “*For example, cinnamic acids exuded by watermelon roots have been documented to inhibit seedling growth while concurrently augmenting the pathogenicity factors of Fusarium oxysporum f. sp. niveum. Coumarins stimulated conidial germination and mycotoxin production of Fon, subsequently inducing a slowdown in plant growth.* ”
**9. Line 92.- the term “autotoxic exudades” means that the plant produces spontaneous cell death and currently this is associated with hypersensitive response, so I would try to present this as and HR produced in response for defense not to be “autotoxic”, the death with pathogens only has sense when this death stop the pathogen and save the plant.**

Thank you for your suggestion. According to a previous study, the root exudates of ginger may inhibit its normal growth by reducing growth parameters, suppressing antioxidant defense enzymes, and decreasing chlorophyll content under certain concentrations (Han et al., 2012b). Therefore, the term "autotoxic" is appropriate. However, to ensure accuracy, we have revised this phrase to "*autotoxic leachates*." We believe that this revised expression is more suitable for the present study.

**10. Lines 95-98.- cite here why you used and selected them, why they come from leaves (see methods) and explain previous specific effects of all of them, here it is necessary to add the steps in which those metabolites are produced by the plant during defense responses to Foz, check previous works including other plant systems and metabolomic studies made of plant systems responding to Foz and against Fol also and determine the sequence and kinetic about how those metabolites are produced by the plant. Probably the kinetic s coordinated and correlated with fungal vital cycle of infection and with penetration into the root.** **Those points has to be considered along the work, as well as into results and conclusion and discussion section. Each metabolite has and specific effect on the fungus because attach the fungus at the different stages of plant-fungal colonization.**

We appreciate your insightful suggestions and guidance. Building on previous research and our own investigations, it has been observed that leachates from various parts of ginger can inhibit normal growth (Han et al., 2012b). Specifically, ferulic acid is the primary component in stem leachate, while syringic acid and 7-hydroxycoumarin are predominant in rhizome leachate. Additionally, coumarin was isolated from root leachate. Consequently, rigorous experiments were conducted to assess the effects of these compounds on ginger growth, revealing their inhibitory potential. These findings provided the preliminary rationale for selecting and employing these compounds in the current study. Accordingly, we have revised and supplemented certain sentences in **line 103, page 5**. “*Therefore, the four main components of ginger leachate from different organs, syringic acid, ferulic acid, coumarin and 7-Hydroxycoumarin, which have been verified to have negative effects on plant growth in our previous study, were employed, and their mechanisms of action were characterized.*”

Numerous studies have demonstrated that plant metabolites significantly influence the outbreak of plant diseases by affecting the growth and activities of phytopathogens and reshaping the rhizosphere microbiome community. However, research on the production of metabolites in ginger during its resistance to *Foz* remains limited. Generally, the accumulation of phenolic acids, such as syringic acid and ferulic acid, at the onset of fungal infection induces plant resistance (Du et al., 2025; Ghorbi et al., 2023). The differentiation between resistant and susceptible cultivars is also based on variations in plant metabolites (Zhang et al., 2020). Therefore, we posit that metabolite accumulation constitutes a defense mechanism during pathogen infection. We will introduce this perspective in the Discussion section and provide a more in-depth and comprehensive interpretation of our results.

**Materials and Methods**

**11. Line 106.- at this article indicates studies in leaves explain and justify in methods why use them.**

Thank you for your suggestion. As previously mentioned, these compounds constitute the primary components of the aqueous extracts from various ginger organs (Han et al., 2012b). Our preliminary investigation also identified their adverse effects on ginger growth. Consequently, these compounds were classified as autotoxic chemicals in the present study. To eliminate the potential influence of impurities, we procured highly purified commercial products. To clarify this, we have revised the corresponding sentences on **line 114, page 6**. “*According to a previous study and our preliminary results, four prevalent metabolites, which have been identified from the aqueous extract of ginger’s organs and have negative effects on growth, were designated as autotoxic chemicals in the present study.*”
**12. Line 112.- Transform this table into text shorter because in all of them the times are the same.**

Thank you. We have deleted Table 1 and transformed their information into the text in **line 129, page 6**. We believe that the present expression can provide more accurate information.
**13. Line 118.- add as follows:**

Thank you. We have revised this part.
**14. Line 122.- why PDA ? how PDA interacts with metabolite structure? Did you check at controls?**

Thank you for your inquiries. It is essential to ascertain the interaction between PDA and the metabolite structure. Based on analogous prior studies, PDA medium was employed as a vector for exposure experiments (Wu et al., 2015; Xu et al., 2025). Our analysis indicated that the PDA medium did not significantly influence the compound content. Consequently, the PDA medium was utilized as the compound vector in the current study.
**15. Lines 156-173.- Did you check if inductions of those plant-genes CWDEs are related to production of certain metabolites ??? check and analyze and add to discussion.**

We appreciate your insightful suggestions, which have been incorporated into the Discussion section (**line 517, page 25**). During pathogen infection, plants accumulate phenolic compounds as a defense mechanism (Mikulic-Petkovsek et al., 2013; Zhang et al., 2022). Concurrently, phytopathogens upregulate the expression of pathogenicity-related genes, such as those encoding cell wall-degrading enzymes (CWDEs), in response to this regulation (Bhagat et al., 2024). This phenomenon elucidates how plant metabolites induce the activity of phytopathogenic CWDEs. We believe that the present interpretation offers a more comprehensive understanding of enzymatic responses to autotoxins.
**16. Line 193.- Not all FUBs are cited into the abstract cite them with specific results. Why only FUB1 and FUB3? Explain at discussion.**

We apologize for any confusion. Our research demonstrated that syringic acid and coumarin significantly induced toxin accumulation, with the *FUB3* response being the most prominent. Therefore, we exclusively referenced *FUB3* in the Abstract. To offer a more precise and concise interpretation of gene expression responses, we revised the description in the Discussion section on **line 549, page 26**, to differentiate the effects of the compounds. Additionally, we have incorporated the gene expression results in the Abstract section on **line 36, page 2**, emphasizing the significance of *FUB3*, *FUB6*, and *FUB9* in our findings.

**17. Line 197.- Why 7 days post treatment? Dis you make previous kinetics? Add and explain.**

Thank you for your inquiries. In an initial experiment, we endeavored to extract total RNA from the liquid medium under identical conditions of FA treatments; however, we encountered challenges in completing this task. Consequently, we opted for PDA medium and mycelia as the materials. Based on preliminary experiments, mycelial growth stabilized after six days of cultivation, and similar results were observed in the gene expression treatments. Furthermore, gene expression may remain unstable until six days post-treatment, potentially leading to variations in the results. Therefore, a duration of seven days post-treatment appears to be appropriate. To eliminate ambiguity, we have included additional sentences in **line 212, page 10**, to clearly convey this information. “*Preliminary experiments indicated that reliable results of gene expression were obtained 7 days post-treatment*.”
**18. Line 210.- cite Livak et al., method for -2 landa CT QRT-PCR calculates. Add here number of replicates and statistical “n” used.**

Thank you. We have added this information to the corresponding sections.

**Statistical**
**19. Specify at each case ANOVA, T-Test, number “n” in all figure legends applied.**

Thank you. We have revised and clarified the information in **Line 252, Page 12**. We believe that the current expression accurately delineates the analysis program.

**Results**

**20. Lines 235, 252,264…etc: please cite at all sections each figure separately.**

We appreciate your recommendations. We have ensured that this information is cited in all sections, with each figure referenced individually.

**21. Line 280, lines 294-295., move to discussion, try to not put conclusions into results section.**

Thank you for your suggestion. We have transformed these sentences into the Discussion section.

**22. Section 334: Consider here to conclude that the most toxic metabolites for the fungus are also inducing defense mycotoxins to stop plant defense response, there is a clear correlation between the induction of FUBs produced by the metabolites and their toxic effects on fungal growth. Please consider this point of view.**

We appreciate your valuable suggestions. Our findings indicate that the metabolites inhibiting the negative growth and reproduction of phytopathogens consistently induced the synthesis of mycotoxins, such as syringic acid, as observed in the present study. Consequently, there may be a correlation between the synthesis of mycotoxins and the growth and reproduction of *Foz*. In **Section 4.4**, we provide a preliminary explanation for this phenomenon. We anticipate that future research on the response of *Foz* metabolic autotoxins will yield a more comprehensive and concise interpretation to support this hypothesis.

**23. Line 337.- consider add those data to abstract.**

Thank you for your suggestion. We have supplemented this data into abstract in **Line 34, Page 2**.
**24. Line 350.- move to abstract and discuss those results.**

We appreciate your suggestion. The sentence has been relocated to both the Abstract and Discussion sections. Additionally, we have included a more comprehensive interpretation in the Discussion section (**Line 586, Page 28**).

**25. Section 356 to 367: This section is just the more important of the article giving very interesting conclusion for me more than that you explained. A pathview program analysis is required to look for correlations between the kinetic of plant response production and metabolism and fungal gene induction and cycle of infection. Consider moving to discussion section except to data obtained.**

We appreciate your suggestion. We have also considered plant metabolites associated with phytopathogen infection. Given that this section emphasizes the comprehensive impact of autotoxins on *Foz*, we have opted to retain this content within the "Results and Analysis" section. Nonetheless, we have enhanced the description in the Discussion section on **line 597, page 28**, to elucidate potential interactions and offer a more detailed explanation.
**26. Here the results described about coumarin probably indicate that coumarin has in plant a direct toxic effect on the fungus reducing the mycelial growth and biomass according to the natural function of coumarin on roots and this very high toxic effect on fungal growth makes that the fungus try to protect itself by producing higher amounts of mycotoxins. This is a combat between not well considered at this section. Similar effects of hydroxycoumarin are according to that and analyze if synergistic effects of both metabolites has been described previously.** **Please consider this point of view making the results of this work much more relevant.**

We express our gratitude for your insightful suggestion. The primary function of plant metabolism is to optimize the growth environment, which encompasses the prevention of pathogen invasions. Our findings indicate that coumarin inhibits pathogen growth while simultaneously stimulating toxin accumulation, a phenomenon that appears to result from plant–pathogen interactions. Consequently, we have proposed a relevant hypothesis (**line 466, page 22**) to elucidate this potential mechanism. We posit that such interactions are not limited to the effects of coumarin but extend to all plant metabolites, and we interpret these interactions in detail in the following sections.

**Discussion**
**26. Lines 370 to 376.- consider for changing the abstract.**

Thank you for your suggestion. We have revised the background part of the Abstract section to clearly express the research background in **line 24, page 2.**
**27. Line 379-381.- Consider that metabolite production is very expensive for the plant, and it will try first to stop the fungus but if this low concentration not work they produce higher quantities, at low concentrations if the compound are very toxic the fungus will try to survive attaching the plant, consider this “combat” at the discussion.**

We appreciate your valuable suggestions. The primary functions of plant metabolites may include defense against phytopathogen infections and facilitation of plant growth. Consequently, the impact of autotoxins on *Foz* should be considered a result of the interaction between plants and pathogens. This information has been incorporated on **line 438, page 21,** and serves as the central theme of the present study.

To elucidate our perspective comprehensively, we have structured the Discussion section into four distinct subsections. The initial subsection is titled "**The allelopathic effect of autotoxins on *Foz* as a consequence of the interaction between ginger's metabolites and pathogens**." We assert that this formulation effectively conveys our findings.
**28. Line 398.- Correlate metabolite production at plants with pathview transcriptional previous data and metabolomics data responding to Fusarium sp.**

Thank you for your suggestion. We have revised the relevant data on **line 460, page 22**, and have employed these studies to substantiate our perspective.
**29. Line 406.- Which are the natural concentrations in plants? Check and explain and discuss here.**

We appreciate your suggestions. Regrettably, there is a paucity of research focusing on the metabolite composition of ginger in situ. Consequently, we have examined analogous data from other plant species and integrated these findings with data from our previous study. Notably, the concentration of phenolic acids in the soil following three years of monoculture was approximately 0.25 mM. This information has been incorporated on **line 479, page 23**.
**30. Line 407-408.- consider that the activity of metabolites will be lower along the time because molecular stability and interaction with PDA medium??**

Thank you for your suggestions. The reduced stimulatory effect of autotoxins may be attributed to several factors. One potential explanation is the interaction between autotoxins and the PDA medium. Alternatively, it may represent one of the response mechanisms of *Foz* to autotoxins, which we consider more plausible. Autotoxins function as abiotic stress factors that regulate the normal growth of pathogens, and the primary role of metabolites may be to defend against pathogen growth and infection. Consequently, the stimulation observed in the present study appears to be a hormesis effect, with the attenuated phenomenon resulting from the acclimation of *Foz*. We anticipate that further studies will be conducted to provide a clearer and more definitive interpretation.

We also added sentences in **line 486, page 23**, to reveal this phenomenon and attributed it to the acclimation of *Foz* to plant regulation.
**31. Lines 413 to 415.- explain clearly, this is not well understood. Explain differences between total biomass and mycelial, most of biomass Is mycelia? Explain. Increasing of mycelial might indicate the high toxic effect of the chemical and activation of fungal metabolism to survive?**

We apologize for any confusion this may have caused. The dry weight of *Foz* consists of mycelia, spores, and their cellular contents. Our findings indicate that high-concentration treatment inhibited mycelial growth, spore production, and germination, while it stimulated biomass accumulation, potentially due to the accumulation of cellular contents. Previous research has documented that phytopathogens can accumulate proteins and enzymes to counteract abiotic stress, such as elevated temperature and osmotic pressure (Zhang et al., 2023). Consequently, we propose that the increased biomass observed in the present study represents the mechanism by which Foz responds to autotoxins. This information has been added on **line 493, page 23**.
**32. Line 421.- explain in detail stimulating process of biomass production.**

Thank you. We have supplemented some sentences in **line 493, page 23**, to interpret biomass production.
**33. Lines 431.- consider the toxic direct effect of coumarin more than the autotoxicity and correspondence with expressions found in FoSir5 gene??**

We appreciate your suggestions. We posit that the stimulatory effect of coumarin is more likely attributable to its special properties rather than autotoxicity. Previous research has demonstrated that plant metabolites can modulate the gene expression of *Fusarium* sp. to mount a defense against infection (Pothiraj et al., 2023). Additionally, the germination of *Foz* spores necessitates overcoming the constraints of aerobic respiration, with the *FoSir5* gene playing a regulatory role in the expression of genes associated with aerobic respiration. Consequently, we propose that the stimulatory effect of coumarin may influence the expression of *FoSir5*. We advocate for further research to elucidate this issue. We have revised the interpretation of this point on **line** **526, page 25**, and assert that the current description offers a more lucid interpretation of our results.
**34. Lines 440 to 455.- Consider the defense responses of the fungus to survive to plant defense production of toxic metabolites associated to infection progress. Contrast your results with previous transcriptional and metabolomic studies made againt Fol and Foz previously, consider the kinetics of fungal penetration and kinetic of plant production of those different metabolites analyzed, I consider that probably you will find very interesting results. Prepare only ONE scheme simpler with the hypothesis to add it into principal article discussion section.**

We are grateful for your insightful suggestions. A prior study has demonstrated that plant metabolites not only regulate the physiological activities of phytopathogens but also recruit beneficial microorganisms to combat plant diseases (Du et al., 2025). Upon infection by pathogenic bacteria, plant root systems secrete substantial quantities of phenolic acids and flavonoids to modulate the growth and development of these pathogens. In response, the pathogens alter their physiological activities to circumvent the plant's defense mechanisms. Consequently, we hypothesize that the impact of autotoxins on enzymes and mycotoxins constitutes a response to plant defense strategies. We have incorporated relevant information on **line 548, page 26**, to elucidate the effect of ginger metabolites on *Foz*.
**35. Line 445.- Hydroxycoumarin does not like to contribute to fungal infection try to get different conclusions according to plant defense and plant-fungal combat. Justify in terms of interaction at nature not on plates. Highlight that PDA is not the same than soil interactions and results should not be extrapolated.**

We apologize for this confusion. Coumarin and 7-Hydroxycoumarin are always defined as defense compounds, although some stimulation effects were recorded in the present study. Consequently, we have revised the conclusion regarding this point and highlighted the interaction with the natural environment in **line 557, page 26**.
**36. Line 474.- consider the protection role of coumarin against the fungus also inhibiting FA synthesis and add to abstract. One of the more interesting results are the coumarin role in plant defense please highlight this into abstract or including into the title.**

We appreciate your suggestion. We have emphasized the defensive role of these compounds in the natural environment and have elucidated the stimulation of coumarin on FA synthesis in **line 592, page 28**. Additionally, we have incorporated this information into the Abstract section.
**37. Lines 486 to 491.- consider that plant is trying to survive attaching the fungus.
Plant metabolites activate cytotoxic fungal responses and for me this is one of the most important results shown at this work and this should be highlighted at title, abstract and conclusions.**

We appreciate your suggestion. As previously indicated, the influence of autotoxins on *Foz* should be understood as the pathogen's response to the regulatory mechanisms of ginger. Consequently, we have taken these factors into account and have drawn new conclusions in **line 622, page 29**. We believe that the current expression clearly articulates our findings.
**388. Line 494.- Consider that this is on plates not at nature but highlight the role of those metabolites in plant for protecting against *Foz* more than increasing the incidence and plant autotoxicity.**

Thank you. We have removed the pertinent sentences and emphasized the defensive roles of these metabolites. We suggest that the enhancing effect of plant metabolites on *Foz* is attributable to the compensatory mechanism of *Foz*; specifically, plant metabolites inhibit the growth or colonization of *Foz*, while *Foz* increases its virulence or pathogenic factors to adapt to the regulatory effects of the host plant. We believe that the current description is appropriate for conveying our findings accurately.
**39. Lines 500 to 503.- I am not according to these conclusions please review and consider changing the conclusions and point of view about very interesting results obtained.**

We appreciate your suggestions. In **line 622 on page 29**, we have formulated a new conclusion that emphasizes the role of metabolites in the defense mechanisms. The influence of ginger metabolites on *Foz* should be interpreted as a response of pathogens to plant regulatory processes. We believe that the revised conclusions offer a more comprehensive explanation of our findings.

**Reference**

Bhagat N, Mansotra R, Patel K, Ambardar S, Vakhlu J. Molecular warfare between pathogenic *Fusarium oxysporum* R1 and host *Crocus sativus* L. unraveled by dual transcriptomics. Plant Cell Rep. 2024;43:42.

Du C Y, Kang Y, Zeng Q, Zhao X Y, Qi G F. Metabolic changes in root exudates of plants with different disease severity affect plant resistance by regulating the rhizosphere microbiome. Pest Manag Sci. 2025;82(2): 1-16.

Ghorbi M, Momeni H, Rashidi V, Ahmadzadeh A, Yarnia M. Changes in Phenolic acid levels in wheat cultivars inoculated with *Pyrenophora tritici-repentis* race 1. J Agr Sci Tech. 2023;25(1): 185-198.

Gordon TR. *Fusarium oxysporum* and the *Fusarium* wilt syndrome. Annu Rev Phytopathol, 2017, 55: 23-39.

Han C, Li C L, Ye S P, Wang H, Pan KH, Wu N. Autotoxic effects of aqueous extracts of ginger on growth of ginger seedings and on antioxidant enzymes, membrane permeability and lipid peroxidation in leaves. Allelopathy J. 2012a;30: 259-270.

Han C, Li C L, Ye S P, Pan K H, Wu N, Li W. Effects of ginger aqueous extract on soil enzyme activity, microbial community structure and soil nutrient content in the rhizosphere soil of ginger seedlings. Acta Ecologica Sinica. 2012b;32(2): 0489-0498．

Mikulic-Petkovsek M, Schmitzer V, Slatnar A, Weber N, Veberic R, Stampar F, et al. Alteration of the content of primary and secondary metabolites in strawberry fruit by Colletotrichum nymphaeae infection. J Agric Food Chem. 2013;61: 5987–5995.

Pothiraj M, Shanmugam V, Tyagi A, Hussain Z, Aggarwal R, Haritha MM, et al. Physiological race characterisation of *Fusarium oxysporum* strains infecting tomato employing candidate pathogenicity genes and host resistance. World J Microb Biot. 2023;39:66.

Zhang ZJ, Sun JL, Zhao SC, Lu QJ, Pan LZ, Zhao BL, et al. Effects of different rootstocks on phenolics in the skin of ‘Cabernet Sauvignon’ and widely targeted metabolome and transcriptome analysis. Hortic Res-England. 2022;9: uhac053.

Zhang MZ, Trushina MK, Lang T, Hahn M, Pasmanik-Chor M, Sharon A. Serine peptidases and increased amounts of soluble proteins contribute to heat priming of the plant pathogenic fungus *Botrytis cinerea*. Fungal Biol-UK. 2023;14(4): 1-19.

**Reviewer #2**

Summary

I have carefully reviewed the manuscript entitled “In vitro Study of the Growth, Reproduction, and Pathogenicity Responses of *Fusarium oxysporum* f. sp. *zingiberi* to Autotoxins from Ginger.” This study is relevant and of potential interest, as it demonstrates that four ginger-related autotoxins differentially affect the growth, reproduction, and virulence-associated traits of *Fusarium oxysporum*, thereby contributing to Fusarium wilt under long-term ginger monoculture. The work further provides mechanistic insight into how these autotoxins may influence disease development. Overall, the manuscript is within the scope of the journal. However, the presentation and writing of the manuscript require substantial improvement, and several important concerns should be addressed before the manuscript can be considered for publication.

**1. Line 1-2 It is recommended that the title be revised to more clearly convey the key message and core theme of the study. The title should present the main focus of the work at a single glance, without requiring the reader to refer to the main text for clarification. In addition, *in vitro* should be italicized in the title. At present, the word “study” is too general and does not clearly reflect the specific message or contribution of the work.**

We appreciate your insightful suggestions. We have revised the title of this research to “**Physiologic and molecular response of *Fusarium oxysporum* f. sp. *zingiberi* to ginger autotoxins**.” We believe that the revised title is more appropriate and concise for this study.

**2. Line 10 The email address of the corresponding author appears to be incorrect. Please provide the correct email address, including the proper “@” symbol and domain name, to ensure accuracy.**

We apologize for any confusion. We have corrected the erroneous email address format in **line 20, page 1**. We believe the current expression meets the requirements of our journal.

**2. Line 23-39 The abstract should be revised to include a clearer problem statement, the existing research gap, and the need or importance of this study in addressing that gap, while more explicitly highlighting the novelty and innovation of the research. In addition, there appears to be an inconsistency in the pathogen name: the title refers to *Fusarium oxysporum* f. sp. *zingiberi*, whereas line 24 mentions *Fusarium oxysporum* f. sp. *niveum* and abbreviates it as Foz. Please verify and correct this for accuracy and consistency throughout the manuscript. The treatment information is also unclear. The term “representative autotoxins” should be defined more precisely, as its meaning is not evident in the current context. Similarly, since the title refers to autotoxins from ginger, it should be clarified whether these compounds were directly extracted from ginger, naturally present in ginger and then applied experimentally, or artificially prepared. The concentrations used in each treatment should also be clearly reported. Furthermore, the result section would benefit from reorganization in a more systematic and mechanistic manner, so that the findings present a clear progression rather than appearing randomly arranged. The results should lead to clear concluding statements that are well aligned with the study objectives, support the title, and emphasize the novelty of the work. Finally, it would strengthen the manuscript to include future perspectives, particularly regarding the scientific significance of the findings and their potential practical benefits for farmers.**

We apologize for any confusion. We have revised the abstract in accordance with our new conclusion. Initially, we emphasized the significance of autotoxins, as explored in this study, as well as the novelty and innovation of our research. Subsequently, the results were presented in a more appropriate and concise manner to substantiate our conclusions. Finally, we underscored the importance of this research and outlined future research directions in the Abstract and Conclusion sections. We believe that the current abstract accurately conveys the content of our study.

**3. Line 99-231 It is suggested that the contents be arranged in a more systematic, stepwise manner so that the methodological details are presented clearly from beginning to end, ensuring reproducibility. You mention that the autotoxins used in this study have been documented previously; if so, the novelty of the present work should be clarified. Please indicate whether these compounds were selected based on prior reports and newly evaluated in a different context, or whether they were simply designated as autotoxins in this study. This point needs to be explained more clearly. In addition, the Table 1 should focus only on the details directly relevant to the present experiment, rather than including doses from other experiments, as this may create confusion. If those doses were optimized in a previous study, there is no need to report the additional concentrations here unless they are directly relevant to the current work. Furthermore, in the methods section, the subsection headings should be written according to methodological procedures rather than beginning with phrases such as “effects of autotoxins on different parameters,” which are more appropriate for the results section. Revising the subsection headings to reflect the actual experimental methods would improve the clarity and organization of the manuscript.**

We apologize for any confusion. We have revised certain expressions in this study to provide additional methodological details and facilitate the replication of our research. The autotoxins utilized in this study have been extensively documented in prior research concerning the adverse effects on ginger growth. However, this study specifically examines the impact of autotoxins on *Foz*. We have included supplementary information in **line 120, page 6**, to elucidate the rationale for designating these compounds as autotoxins in this study.

We have also added supplementary sentences to elucidate the specifics of the enzyme activity treatments, as indicated in **line 167 on page 8**. “*To examine the influence of autotoxic compounds on enzymatic activity, five agar plugs obtained from a 7-day cultivation were transferred to a liquid medium and incubated at 28℃ for seven days under shaking conditions at 180 rpm. The resulting broth was filtered through four layers of sterile gauze and utilized as a crude enzyme preparation.*”

We have removed Table 1 and incorporated the information into the text on **line 129, page 6.** The dosages employed in the current study were derived from the findings of our preliminary experiments. Our results indicated that mycelial growth, spore germination, and sporulation demonstrated varying sensitivities to these compounds. Consequently, different autotoxin components were utilized in these investigations. We have now corrected the inaccuracies and provided a more precise account of the methodological details.

We have amended the erroneous subtitle in the Materials and Methods section. The revised subtitles now accurately represent the experimental methodologies employed.

**Line 232-367 In the results section, the subheadings should be revised to be concise, informative, and sufficiently descriptive of the main content presented under each subsection. Clear and well-structured subheadings would improve the organization of the section and help guide the reader through the key findings more effectively.**

We appreciate your suggestions. Initially, we intended to employ the terms "inhibition" or "stimulation" to characterize the respective sections. However, given that different autotoxins exerted varying effects on *Foz*, we opted to use the term "effect" to describe these sections. We have revised the subtitle to enhance clarity and structure, and we believe that the current description offers a more appropriate expression.

**Line 368 503 The discussion section would benefit from being divided into clearly defined subsections, with the content organized under appropriate headings for improved clarity and structure. This section should emphasize the major findings of the study and provide a deeper interpretation of the morphological, biochemical, physiological, and molecular mechanisms underlying the observed responses. Moreover, the conclusion should be presented as a separate and independent section, as commonly required by many journals. In the present manuscript, the conclusion appears to be incorporated within the discussion section (lines 491–503), and this should be revised in accordance with the journal’s formatting guidelines. The conclusion section should summarize the main findings in a systematic and mechanistic manner and lead to a clear, strong concluding statement that directly supports the study objectives and research hypothesis, while remaining well aligned with the title of the manuscript. In addition, the authors are encouraged to acknowledge the limitations of the study, outline future research perspectives, and highlight the practical significance of the work for both the scientific community and farmers.**

We appreciate your insightful suggestions. Initially, we introduced appropriate headings to enhance the structure and clarity of the content. We have revised and integrated certain sentences to offer a more comprehensive interpretation of our findings. We believe that the current description provides a clearer and more precise explanation of the results.

In accordance with the journal's requirements, we have established the conclusion as a distinct and independent section. Additionally, we have revised the content of the conclusion to incorporate new perspectives derived from our findings. We believe that the current conclusion more effectively summarizes and supports our results.

In conclusion, we have incorporated information to delineate the limitations of the current study and suggest potential avenues for future research in **Line 622, Page 29.** Additionally, we have emphasized the practical implications of this study for both the scientific community and agricultural practitioners at the conclusion of the Discussion section.

**Line 505-693 The references should be thoroughly checked and revised to ensure full compliance with the journal’s formatting guidelines. At present, there are several inconsistencies in reference style. For instance, the journal name appears in uppercase letters in lines 517–518, while journal names are abbreviated in most other entries; however, in line 564, the journal name is provided in full. These inconsistencies should be corrected to maintain accuracy, uniformity, and adherence to the journal’s required format.**

We apologize for any confusion. We have revised the style and format of the references in the current study to ensure consistency in the reference list. However, some journals lack officially designated abbreviated names; therefore, full names were used in this study. We are confident that this edition satisfies the requirements of our journal.
